# Association of symptomatic gallstones and primary hyperparathyroidism: a propensity score-matched analysis

**DOI:** 10.1093/bjs/znab249

**Published:** 2021-08-09

**Authors:** Y Saito, H Takami, A H Abdelhamid Ahmed, A Nakao, K Ho, T Tokuda, R Miyata, G W Randolph, N Ando

**Affiliations:** Department of Surgery, International Goodwill Hospital, Yokohama, Kanagawa, Japan; Department of Surgery, Keio University School of Medicine, Tokyo, Japan; Department of Otolaryngology, Massachusetts Eye and Ear Infirmary, Boston, Massachusetts, USA; Department of Surgery, Ito Hospital, Tokyo, Japan; Department of Otolaryngology, Massachusetts Eye and Ear Infirmary, Boston, Massachusetts, USA; Department of Surgery, International Goodwill Hospital, Yokohama, Kanagawa, Japan; Department of Surgery, International Goodwill Hospital, Yokohama, Kanagawa, Japan; Department of Surgery, International Goodwill Hospital, Yokohama, Kanagawa, Japan; Department of Surgery, International Goodwill Hospital, Yokohama, Kanagawa, Japan; Department of Otolaryngology, Massachusetts Eye and Ear Infirmary, Boston, Massachusetts, USA; Department of Surgery, International Goodwill Hospital, Yokohama, Kanagawa, Japan

## Abstract

If the prevalence of primary hyperparathyroidism (PHPT) in patients with symptomatic gallstones is higher than that in the general population, PHPT screening could reveal important clinical implications. We observed that the prevalence of PHPT in these patients was higher compared to that of healthy matched controls.

*Dear Editor*

Gallstones may be a complication related to primary hyperparathyroidism (PHPT)^1^^,^^2^ but the prevalence is not known. Screening could reveal important clinical implications if the prevalence of PHPT is higher than that of the general population. This retrospective propensity score-matched study evaluated the association between symptomatic gallstones and PHPT .

Data were collected from April 2012 to May 2020 at International Goodwill Hospital, Kanagawa, Japan (approved by the ethics committee). Patients who underwent a cholecystectomy for symptomatic gallstones were compared with healthy matched controls who underwent a general health screening at the hospital during the same period. The exclusion criterion was renal insufficiency (a serum creatinine level greater than 1.47 mg/dl). Patients who underwent a cholecystectomy at the time of another operation (for example, gastric surgery) and those with gallbladder tumours, adenomyosis, asymptomatic gallstones or acalculous cholecystitis were excluded.

The primary outcome measure was the rate of hypercalcaemia. Because screening rates of PHPT in hypercalcaemic patients typically remain surprisingly low^3^^,^^4^, a predicted rate of PHPT was used. PHPT and malignancy account for more than 90% of hypercalcaemia cases^5^. The predicted number of PHPT cases was calculated by subtracting the number of patients with malignancy from 90 per cent of the number of patients with hypercalcaemia. Increasing age, female sex and obesity are well known risk factors for gallstones, and women around menopause and with higher body weight appear to be at particular risk of PHPT. A 1 : 1 propensity score matching was performed to reduce selection bias. Pearson's chi-squared test was used for the comparison of categorical variables. Student's t-test and the Mann-Whitney U-test were used to compare continuous variables. For all procedures, p-values <0.05 were considered significant.

During the 8-year period, 930 patients underwent a cholecystectomy, and 2219 individuals underwent the general health screening. After exclusions, 709 and 2211 patients were included in the symptomatic gallstone and control groups, respectively. After matching, 702 patients were extracted from each group. The two groups were well matched for key confounders, that is age, sex, BMI and serum creatinine level (*Table 1*). Compared with the controls, the symptomatic gallstone group's rate of hypercalcaemia (0.4 *versus* 2.3 per cent; *P* = 0.003), rate of diagnosed PHPT (0 *versus* 0.7 per cent; *P* = 0.025) and predicted rate of PHPT (0.4 *versus* 2.1 per cent; *P* = 0.004) were significantly higher. Among the patients with hypercalcaemia, 43.8 per cent underwent a PHPT screening test. It is interesting to note that 80 per cent of the PHPT patients with symptomatic gallstones needed to undergo a parathyroidectomy for other surgical indications.

**Table 1:** Comparison of characteristics and outcomes between the control and symptomatic gallstone groups before and after propensity score matching

	Before propensity score-matching			After propensity score-matching		
	**Control** **(*n* = 2211)**	**Symptomatic gallstone** **(*n* = 709)**	*P*	**Control** **(*n* = 702)**	**Symptomatic gallstone** **(*n* = 702)**	***P***
**Baseline characteristics**						
Age (years)*	64 (56–71)	67 (55–75)	<0.001	66 (59–72)	67 (55–75)	0.428
Gender			0.001			0.708
Male	1326 (60)	377 (53)		369 (53)	376 (54)	
Female	885 (40)	332 (47)		333 (47)	326 (46)	
BMI (kg/m^2^)*	22.3 (20.3–24.4)	23.8 (21.1–26.1)	<0.001	23.4 (21.3–25.9)	23.8 (21.1–25.7)	0.255
Serum creatinine level (mg/dl)*	0.77 (0.65–0.89)	0.74 (0.63–0.89)	0.011	0.74 (0.63–0.87)	0.74 (0.63–0.89)	0.757
History of cholecystectomy				–	40 (5.7)	–
Gallstones detected by AUS				–	55 (7.8)	–
**Outcomes**						
Hypercalcaemia				3 (0.4)	16 (2.3)	0.003
Albumin-corrected calcium level*				9.4 (9.2– 9.6)	9.4 (9.2–9.7)	0.066
Diagnosed PHPT				0 (0)	5 (0.7)	0.025
Predicted PHPT				2.7 (0.4)	14.4 (2.1)	0.004

Values in parentheses are percentages unless indicated otherwise; *values are median (i.q.r.). Control group: individuals who underwent a general health screening. Symptomatic gallstone group: patients who underwent a cholecystectomy for symptomatic gallstone disease. A serum calcium concentration of 10.4 mg/dl (2.60 mmol/l) was chosen as the cut-off for hypercalcaemia. All of the controls had provided their medical history and undergone an abdominal ultrasound (AUS) examination. PHPT, primary hyperparathyroidism

The prevalence of hypercalcaemia and the predicted prevalence of PHPT in the patients with symptomatic gallstones were both over five-fold higher than those for the healthy matched individuals. Although it is unclear whether a parathyroidectomy is indicated in PHPT patients simply because they have symptomatic gallstones, most of the PHPT patients with symptomatic gallstones had other surgical indications.

The pathogenesis of gallstones in PHPT remains unclear. Possible mechanisms may involve gallbladder stasis and a modification of bile composition. Because hypercalcaemia is known to decrease the body's bile flow and increase the biliary ionized calcium concentration, gallbladder stasis may lead to an increase in bile concentration, precipitation of cholesterol and calcium salts, retention of biliary precipitates and maturation of gallstones^2^. Some study limitations should be noted: normocalcaemic PHPT was not investigated, and the prevalence of both PHPT and gallstones varies globally. Conducting the proper checks on serum albumin and calcium in routine preoperative blood tests in patients with symptomatic gallstones could reduce the risks of PHPT-related diseases that are associated with overlooked or untreated PHPT.

*Disclosures.* The authors declare no conflict of interest.
